# *KCNJ11* variants and their effect on the association between serum potassium and diabetes risk in the Atherosclerosis Risk in Communities (ARIC) Study and Jackson Heart Study (JHS) cohorts

**DOI:** 10.1371/journal.pone.0203213

**Published:** 2018-08-31

**Authors:** Ranee Chatterjee, Clemontina A. Davenport, Laura M. Raffield, Nisa Maruthur, Leslie Lange, Elizabeth Selvin, Kenneth Butler, Hsin-Chieh Yeh, James G. Wilson, Adolfo Correa, David Edelman, Elizabeth Hauser

**Affiliations:** 1 Duke University, Durham, NC, United States of America; 2 University of North Carolina, Chapel Hill, NC, United States of America; 3 Johns Hopkins University,Baltimore, MD, United States of America; 4 University of Colorado, Denver,CO, United States of America; 5 University of Mississippi Medical Center, Jackson, MS, United States of America; Case Western Reserve University, UNITED STATES

## Abstract

**Background:**

In the Atherosclerosis Risk in Communities (ARIC) Study and Jackson Heart Study (JHS) cohorts, serum potassium (K) is an independent predictor of diabetes risk, particularly among African-American participants. Experimental studies show that serum K levels affects insulin secretion. The *KCNJ11* gene encodes for a K channel that regulates insulin secretion and whose function is affected by serum K levels. Variants in *KCNJ11* are associated with increased diabetes risk. We hypothesized that there could be a gene-by-environment interaction between *KCNJ11* variation and serum K on diabetes risk.

**Methods:**

Evaluating a combined cohort of ARIC and JHS participants, we sought to determine if *KCNJ11* variants are risk factors for diabetes; and if *KCNJ11* variants modify the association between serum K and diabetes risk. Among participants without diabetes at baseline, we performed multivariable logistic regression to determine the effect of serum K, *KCNJ11* variants, and their interactions on the odds of incident diabetes mellitus over 8–9 years in the entire cohort and by race.

**Results:**

Of 11,812 participants, 3220 (27%) participants developed diabetes. 48% and 47% had 1 or 2 diabetes risk alleles of rs5215 and rs5219, respectively. Caucasians had higher proportions of these risk alleles compared to African Americans (60% vs 17% for rs5215 and 60% vs 13% for rs5219, p<0.01). Serum K was a significant independent predictor of incident diabetes. Neither rs5215 nor rs5219 was associated with incident diabetes. In multivariable models, we found no statistically significant interactions between race and either rs5215 or rs5219 (*P*-values 0.493 and 0.496, respectively); nor between serum K and either rs5215 or rs5219 on odds of incident diabetes (*P*-values 0.534 and 0.687, respectively).

**Conclusion:**

In this cohort, rs5215 and rs5219 of *KCNJ11* were not significant predictors of incident diabetes nor effect modifiers of the association between serum K and incident diabetes.

## Introduction

Prior analyses of the Atherosclerosis Risk in Communities (ARIC) Study and Jackson Heart Study (JHS) cohorts have shown low-normal serum potassium (K) to be associated with risk of diabetes, independent of traditional risk factors [1, 2], and that this association is more robust among African Americans than Caucasians [3, 4]. The mechanism for this association remains to be elucidated. However, past experimental studies conducted in healthy adults revealed that induction of low serum K, through use of high-dose diuretics or low-potassium diet and laxatives, can lead to the development of impaired glucose tolerance, through impairments in insulin secretion from pancreatic β-cells [5–7].

Many genetic variants are associated with increased diabetes risk, and some of these genetic variants reside in or near genes encoding voltage-gated K channels of pancreatic β-cells integral to glucose-stimulated insulin secretion [8]. These K-channels are stimulated by circulating glucose levels and allow entry of serum K into the cell. The subsequent high K environment leads to channel closure and stimulates insulin secretion. The genes encoding these channels include *KCNJ11*, *KCNQ1*, and *ABCC8* [8, 9]. Two variants in *KCNJ11*, rs5215 and rs5219, are associated with an increased risk of type 2 diabetes in Caucasians and East Asians [10]; and rs5215 has also been found to be a significant predictor of type 2 diabetes in African Americans [11]. Variants in K channel genes have been associated with functional changes [9], and serum K levels could impact the function of these channels differentially based on variations in the channels.

A previous study has evaluated the significance of certain genetic variants on diabetes risk in the entire ARIC cohort; however, this study did not include the rs5219 *KCNJ11* variant [11]. There have been no studies to determine the impact of the *KCNJ11* variants on diabetes risk in the JHS cohort. Direct genotyping or good quality imputation data for *KCNJ11* rs5215 and rs5219 were available in both ARIC and JHS cohorts; genotype data for risk alleles of *KCNQ1* and *ABCC8* were not available in both cohorts.

In this study, we combined data from the ARIC and JHS cohorts to determine: 1) if there is an association between *KCNJ11* variants (rs5215 and rs5219) and risk of incident type 2 diabetes; 2) if these genetic variants are significant effect modifiers of the association between serum K and incident diabetes risk; and 3) if these associations and interactions differ by race. We hypothesized that the potential impact of low-normal serum K on incident diabetes risk may be stronger among people with an increased risk of diabetes due to *KCNJ11* variants. If an interaction exists between the presence of these genetic variants and serum K levels, then interventions designed to increase serum K levels may preferentially help to decrease incident diabetes risk in people with these genetic variants.

## Materials and methods

Institutional review boards at each of the participating institutions approved the study. In particular, the Duke University IRB approved this study as exempt research. The ARIC study is a prospective cohort of 15,792 adults between the ages of 45 and 64 years at the baseline visit. Participants were recruited from four communities in the US—Forsyth County, North Carolina, Jackson, Mississippi, Minneapolis, Minnesota, and Washington County, Maryland. All participants from Jackson, MS were African-American, while participants from Forsyth County included both African Americans and Caucasians. The participants in the other two communities were primarily white. All participants attended an initial baseline visit between 1987–1989. Three subsequent in-person visits took place at approximately three-year intervals for nine years of follow-up. Details of the design and conduct of the ARIC study have been published previously [12]. JHS is a community-based, prospective cohort study of 5,306 African-American adults, aged 21–95 years at the baseline visit, from the Jackson, Mississippi metropolitan area. Participants had baseline data collected between 2000 and 2004. Approximately 30% of the participants had participated in the ARIC study; the rest of the cohort was recruited from community-sampling approaches, volunteers, and families of other JHS participants [13]. Participants came for in-person visits approximately every four years and had telephone follow-up annually, through 2012, for an average of eight years of follow-up. Institutional review boards at each of the participating institutions approved the study.

### Study participants

**Fig 1:**
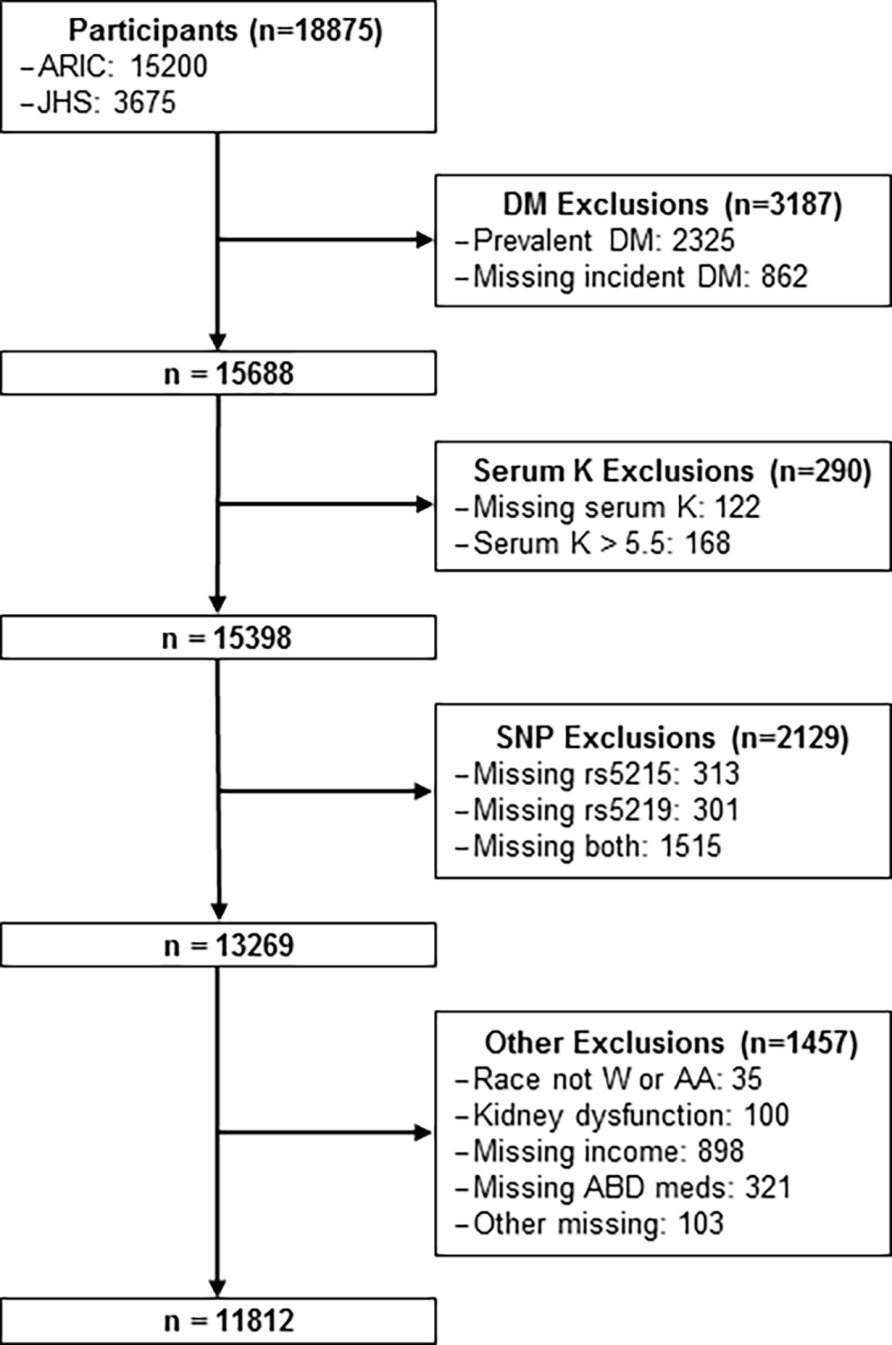
Flow diagram of study participants.

We excluded 592 ARIC participants who had not fasted 8 or more hours for the baseline visit; all participants from JHS had fasted 8 or more hours for their baseline visit. The remaining, non-overlapping ARIC and JHS study populations contained 18,875 individuals. Of these participants, we excluded participants who had prevalent diabetes (defined below) at baseline exam (N = 2325); missing information on diabetes status at baseline or any follow-up (N = 862); serum K > 5.5 (N = 168) or missing information on serum K (N = 122); missing SNP data (N = 2129), evidence of kidney dysfunction (eGFR < 30 mL/min/1.73m^2^ or serum creatinine > 1.7 mg/dL) (N = 100); race other than black/African American or white/Caucasian (N = 35); and missing other covariates (N = 1322). This left a total of 11,812 participants (27% African American) for analyses (**Fig 1**).

### Main exposures

Genotype data for the *KCNJ11* variants rs5215 and rs5219 were available in both the ARIC and JHS cohorts. Both cohorts used the same genotyping methods which were performed at the Broad Institute, Boston, MA. In the ARIC cohort, these variants were determined by direct genotyping using the Affymetrix Genome-Wide Human SNP Array 6.0 (Affy6.0) according to the manufacturer’s recommendations. Further details regarding the genotyping protocol including quality control have been published previously [14, 15]. In the JHS cohort, these variants were either genotyped directly (rs5215, call rate 0.868); or imputed from genome wide genotyping (Affymetrix 6.0) using the 1000 Genomes cosmopolitan reference panel (phase 1 v3) (rs5219, imputation Rsq 0.985) [16]. The two variants were coded as the number of diabetes risk alleles and kept as a continuous variable in the main analyses and dichotomized into carriers (at least one minor allele) and non-carriers (no minor alleles) in sensitivity analyses. The diabetes risk allele for r5215 was assumed to be C; the diabetes risk allele for r5219 was assumed to be T.

Serum K (mmol/L) was measured at the baseline exam with a direct electrode potentiometric assay on undiluted serum. For the main analyses, this measure was categorized into quartiles (with the highest quartile as the reference group). In sensitivity analyses, serum K was analyzed as a continuous variable, scaled by its standard deviation.

### Outcome

The outcome of interest was incident diabetes during the 8 or 9 years of follow-up. A diagnosis of diabetes was based on one of the following criteria: 1) self-report history of diabetes ("Has a doctor ever said you had diabetes?"); 2) fasting glucose ≥ 126 mg/dL; 3) use of diabetic medication (actual and/or self-reported) within 2 weeks prior to clinic visit; or 4) hemoglobin A1c (A1c) ≥ 6.5% (for JHS participants only). Incident diabetes was defined as evidence of diabetes based on the criteria above at any follow-up visit.

### Covariates

For these analyses, all covariates were derived from the baseline exams, which took place in 1987–1989 for ARIC participants and in 2000–2004 for JHS participants. Covariates were chosen based on their potential association with diabetes risk and serum K levels. The following covariates were based on self-report: age (years), modeled as a continuous variable; sex, female or male; self-reported race, categorized as white or black, with white used as the reference category; family income, categorized into 4 groups, <12k, 12k to <25k, 25k to <50k, and ≥50k, with lowest income category used as the reference; parental history of diabetes, self-report of either parent having a history of diabetes. Other covariates included variables determined by measurements at the in-person visits as follows: body mass index (BMI) (kg/m^2^); systolic blood pressure (mmHg); presence of hypertension: per Joint National Committee (JNC) 7, hypertension is defined as blood pressure ≥ 140/90 or self-report use of blood pressure-lowering medication within 2 weeks prior to clinic visit; leisure index: a 4 item questionnaire with 5 Likert-type responses was used to measure the amount of physical activity during leisure time. The index is the average score of the 4 questions and ranges from 1 to 5, where a higher score means more active and a lower score means more leisurely. The following variables were analyzed from biological specimens collected during the baseline visit: serum creatinine (mg/dL); serum sodium (mmol/L); fasting plasma glucose (mg/dL); and fasting plasma insulin (IU/mL). Laboratory assays used for these measurements in both cohorts have been published previously [12, 17, 18]. The use of the following medications, taken individually or in a combination form, was verified by in-person assessment of medication bottles at the baseline visit: use of diuretics, β-blockers, angiotensin-converting enzyme (ACE) inhibitors, or angiotensin II receptor antagonists.

### Statistical methods

Baseline demographic and clinical characteristics of study participants were examined descriptively, by race/ethnicity. Exploratory logistic regression models were fit to investigate interaction effects of race and *KCNJ11* variants (SNP) on the odds of incident diabetes. A likelihood-ratio test was used to determine if the race X SNP term had significant impact on odds of incident diabetes. Logistic regression was performed with minimally-adjusted and fully-adjusted multivariable models to investigate the association between incident diabetes and serum K, SNP, and serum K X SNP, respectively. A likelihood-ratio test was used to determine if the K X SNP term had significant impact on odds of incident diabetes. When these interaction terms were found not to be statistically significant, we ran the models without the interaction terms. Models were adjusted for age, sex, and race (minimally-adjusted, Model 1); additionally with either serum K or the SNP of interest (minimally-adjusted, Model 2). For multivariable models, additional adjustments were made for income, BMI, systolic blood pressure, serum creatinine, serum sodium, fasting plasma glucose, fasting plasma insulin, hypertension, parental history of diabetes, physical activity, and use of diuretics or β-blockers or ACE inhibitors, or Angiotensin II receptor antagonists, (fully-adjusted, Model 3); additionally with either serum K or the SNP of interest (fully-adjusted, Model 4).

We performed several sensitivity analyses to determine if inferences were sensitive to different assumptions. We performed analyses to determine if the inference was sensitive to serum K as a continuous variable or to combining the minor allele categories. We also examined associations within sub-groups of our total cohort. In the JHS sub-group, we performed analyses adjusting for known pedigrees in the JHS cohort. Additionally, in each sub-group, ARIC and JHS, we performed analyses adjusting for population substructure variables available for each study.

Statistical analyses were performed using R 3.3.0 (R Core Team 2016, Vienna, Austria) and SAS 9.4 (SAS Institute, Cary, NC). All hypothesis tests were 2-sided at the 0.05 level of significance. No adjustment for multiple testing for the 2 SNPs was performed.

## Results

### Study population

Based on eligibility criteria described above, we included 11,812 participants in the models for the overall cohort, of which 8,653 were Caucasian and 3,159 were African American. Fig 1 depicts the selection of participants for these analyses. Baseline characteristics of these participants are shown by race/ethnicity in **Table 1**. Compared to Caucasians, on average African Americans had a higher BMI, higher blood pressure and prevalence of hypertension, higher prevalence of a parental history of diabetes, less physical activity, lower income, and lower serum K.

**Table 1:** Baseline characteristics of 11,812 ARIC and JHS participants overall and stratified by race.

	Overall	Caucasians	African Americans
**n**	11,812	8653	3159
**Age (years)**	53.6 ± 6.3	54.1 ± 5.7	52.3 ± 7.6
**Female sex**	6631 (56.1)	4642 (53.7)	1989 (63.0)
**Field center**			
**Forsyth County, NC**	2945 (24.9)	2686 (31.0)	259 (8.2)
**Jackson, MS**	2865 (24.3)	0 (0)	2865 (90.7)
**Minneapolis, MN**	2957 (25.0)	2944 (34.0)	13 (0.4)
**Washington County, MD**	3045 (25.8)	3023 (34.9)	22 (0.7)
**Income**			
**<$12,000**	1396 (11.8)	496 (5.7)	900 (28.5)
**$12,000 to < $25,000**	2487 (21.1)	1619 (18.7)	868 (27.5)
**$25,000 to < $50,000**	4543 (38.5)	3743 (43.3)	800 (25.3)
**≥ $50,000**	3386 (28.7)	2795 (32.3)	591 (18.7)
**BMI (kg/m**^**2**^**)**	27.5 ± 5.4	26.7 ± 4.6	29.9 ± 6.5
**Activity**	2.4 ± 0.6	2.5 ± 0.5	2.1 ± 0.7
**eGFR (mL/min/1.73 m**^**2**^**)**	71.7 ± 14.4	67.9 ± 10.6	82.1 ± 17.8
**Serum creatinine (mg/dL)**	1.1 ± 0.2	1.1 ± 0.2	1.1 ± 0.2
**Serum sodium (mmol/L)**	141 ± 2.3	140.9 ± 2.3	141.2 ± 2.4
**Fasting plasma glucose (mg/dL)**	97.9 ± 9.5	98.5 ± 9.0	96.1 ± 10.5
**Fasting plasma insulin (IU/mL**)	11.5 ± 8.5	10.4 ± 7.6	14.5 ± 10.0
**Systolic blood pressure (mmHg)**	119.8 ± 17.7	117.5 ± 16.6	126.0 ± 19.2
**ABD medications**^**a**^	2779 (23.5)	1767 (20.4)	1012 (32.0)
**Hypertension**	3773 (31.9)	2142 (24.8)	1631 (51.6)
**Parent history of diabetes mellitus**	2567 (21.7)	1730 (20.0)	837 (26.5)
**Serum potassium (quartiles) (mmol/L)**			
**2.5 ≤ K < 4.1**	2314 (19.6)	1245 (14.4)	1069 (33.8)
**4.1 ≤ K < 4.4**	3157 (26.7)	2124 (24.6)	1033 (32.7)
**4.4 ≤ K < 4.7**	2946 (24.9)	2336 (27.0)	610 (19.3)
**4.7 ≤ K < 5.5**	3395 (28.7)	2948 (34.1)	447 (14.2)
**Serum potassium (continuous) (mmol/L)**	4.4 ± 0.5	4.5 ± 0.4	4.2 ± 0.4
**rs5215**			
**0 diabetes risk alleles**	6088 (51.5)	3468 (40.1)	2620 (82.9)
**1 diabetes risk allele**	4461 (37.8)	3949 (45.6)	512 (16.2)
**2 diabetes risk alleles**	1263 (10.7)	1236 (14.3)	27 (0.9)
**rs5219**			
**0 diabetes risk alleles**	6217 (52.6)	3466 (40.1)	2751 (87.1)
**1 diabetes risk allele**	4355 (36.9)	3963 (45.8)	392 (12.4)
**2 diabetes risk alleles**	1240 (10.5)	1224 (14.2)	16 (0.5)

Continuous variables presented as mean ± SD

Categorical variables presented as n (%)

^**a**^ABD medications to include use of diuretics or β-blockers or ACE inhibitors, or Angiotensin II receptor antagonists

The presence of 1 and 2 diabetes risk allele(s) of rs5215 was found in 38% and 11% of the entire cohort, respectively; while the presence of 1 and 2 diabetes risk allele(s) of rs5219 was found in 37% and 11% of the entire cohort, respectively. Only 17% and 13% of African American participants had 1 or 2 minor alleles for rs5215 and rs5219, respectively. Among Caucasian participants, 60% had 1 or 2 minor alleles for rs5215, and 60% had 1 or 2 minor alleles for rs5219 (Table 1). In this cohort, 3220 (27%) participants developed diabetes during the follow-up period.

### Interaction results

In fully-adjusted multivariable models, there was no significant interaction between race and either rs5215 or rs5219 (*P* = 0.49 and 0.50, respectively) on odds of incident diabetes. In the fully-adjusted multivariable models, there was no significant interaction between serum K and either rs5215 or rs5219 on odds of incident diabetes (*P* = 0.54 and 0.69, respectively) (**Table 2**).

**Table 2:** Interaction effects of serum K and respectively, rs5215 and rs5219 on incident diabetes in 11,812 ARIC-JHS participants, overall and by race.

	Overall	P	Whites	P	Blacks	P
Minimally Adjusted, Model 1	OR (95% CI)		OR (95% CI)		OR (95% CI)	
**Serum K X rs5215**						
**2.5 ≤ K < 4.1**	0.88 (0.73, 1.05)	0.162	0.88 (0.71, 1.1)	0.259	1.05 (0.57, 1.91)	0.884
**4.1 ≤ K < 4.4**	1.03 (0.87, 1.21)	0.745	1.03 (0.85, 1.24)	0.781	1.26 (0.69, 2.3)	0.446
**4.4 ≤ K < 4.7**	1.01 (0.85, 1.19)	0.910	0.96 (0.8, 1.16)	0.691	1.15 (0.58, 2.28)	0.698
**4.7 ≤ K ≤ 5.5**	1 (ref)		1 (ref)		1 (ref)	
**Serum K X rs5219**						
**2.5 ≤ K < 4.1**	0.89 (0.74, 1.07)	0.223	0.9 (0.72, 1.12)	0.325	0.89 (0.46, 1.74)	0.734
**4.1 ≤ K < 4.4**	1.04 (0.88, 1.23)	0.637	1.05 (0.87, 1.27)	0.610	1.07 (0.56, 2.06)	0.830
**4.4 ≤ K < 4.7**	1.02 (0.86, 1.2)	0.826	0.97 (0.81, 1.16)	0.737	1.11 (0.53, 2.33)	0.782
**4.7 ≤ K ≤ 5.5**	1 (ref)		1 (ref)		1 (ref)	
**Fully Adjusted, Model 2**						
**Serum K X rs5215**						
**2.5 ≤ K < 4.1**	0.9 (0.73, 1.09)	0.279	0.84 (0.66, 1.07)	0.169	1.09 (0.57, 2.11)	0.788
**4.1 ≤ K < 4.4**	1.03 (0.86, 1.23)	0.773	0.99 (0.8, 1.21)	0.900	1.42 (0.73, 2.74)	0.302
**4.4 ≤ K < 4.7**	1.03 (0.85, 1.23)	0.789	0.98 (0.8, 1.2)	0.834	1.25 (0.59, 2.66)	0.554
**4.7 ≤ K ≤ 5.5**	1 (ref)		1 (ref)		1 (ref)	
**Serum K X rs5219**						
**2.5 ≤ K < 4.1**	0.92 (0.75, 1.12)	0.404	0.85 (0.67, 1.09)	0.194	0.9 (0.43, 1.89)	0.777
**4.1 ≤ K < 4.4**	1.03 (0.86, 1.23)	0.757	1 (0.82, 1.23)	0.974	1.1 (0.53, 2.26)	0.799
**4.4 ≤ K < 4.7**	1.03 (0.86, 1.23)	0.776	0.98 (0.8, 1.2)	0.843	1.15 (0.51, 2.59)	0.743
**4.7 ≤ K ≤ 5.5**	1 (ref)		1 (ref)		1 (ref)	

**Model 1-** adjusted for age, sex, race (overall only), SNP (rs5215 or rs5219), K, SNP X K

**Model 2-** adjusted for age, sex, race (overall only), BMI, systolic blood pressure, presence of hypertension, use of ABD medications, serum creatinine, serum sodium, fasting glucose, fasting insulin, parental history of diabetes mellitus, physical activity, income, SNP (rs5215 or rs5219), K, SNP X K

### Models 1 and 2, minimally-adjusted multivariable models

In minimally-adjusted models, there was a significant inverse association between serum K and incident diabetes. Among all participants, after adjusting for age, sex, and race, those in the lowest serum K quartile had significantly higher odds of incident diabetes compared to those in the highest serum K quartile [OR = 1.39, 95% CI = (1.23, 1.57)]. When stratified by race, results were similar for both whites and blacks (Model 1, **Table 3**).

**Table 3:** Associations of serum K, rs5215, and rs5219 with incident diabetes in 11,812 ARIC-JHS participants, overall and by race.

Minimally Adjusted, Model 1	Overall	P-value	Whites	P- value	Blacks	P- value
Serum K (mmol/L)	OR (95% CI)		OR (95% CI)		OR (95% CI)	
**2.5 ≤ K < 4.1**	1.39 (1.23, 1.57)	0.000	1.36 (1.17, 1.58)	0.000	1.37 (1.08, 1.75)	0.010
**4.1 ≤ K < 4.4**	1.18 (1.06, 1.32)	0.004	1.18 (1.04, 1.34)	0.012	1.17 (0.91, 1.49)	0.217
**4.4 ≤ K < 4.7**	1.12 (1, 1.25)	0.059	1.16 (1.02, 1.32)	0.021	0.97 (0.74, 1.27)	0.827
**4.7 ≤ K ≤ 5.5**	1 (ref)		1 (ref)		1 (ref)	
**rs5215**	0.98 (0.92, 1.05)	0.563	0.99 (0.92, 1.06)	0.794	0.92 (0.76, 1.11)	0.386
**rs5219**	0.99 (0.92, 1.06)	0.732	0.99 (0.92, 1.06)	0.702	1.02 (0.82, 1.25)	0.887
**Minimally Adjusted, Model 2 with both K and SNP**						
**Serum K (mmol/L)**						
**2.5 ≤ K < 4.1**	1.39 (1.23, 1.57)	0.000	1.36 (1.17, 1.58)	0.000	1.37 (1.08, 1.75)	0.010
**4.1 ≤ K < 4.4**	1.18 (1.06, 1.32)	0.004	1.18 (1.04, 1.34)	0.012	1.17 (0.91, 1.49)	0.216
**4.4 ≤ K < 4.7**	1.12 (1, 1.25)	0.060	1.16 (1.02, 1.32)	0.022	0.97 (0.74, 1.27)	0.823
**4.7 ≤ K ≤ 5.5**	1 (ref)		1 (ref)		1 (ref)	
**rs5215**	0.98 (0.92, 1.05)	0.569	0.99 (0.92, 1.06)	0.810	0.92 (0.76, 1.11)	0.382
**Serum K (mmol/L)**						
**2.5 ≤ K < 4.1**	1.39 (1.23, 1.57)	0.000	1.36 (1.17, 1.58)	0.000	1.37 (1.08, 1.75)	0.010
**4.1 ≤ K < 4.4**	1.18 (1.06, 1.32)	0.004	1.18 (1.04, 1.34)	0.012	1.17 (0.91, 1.48)	0.217
**4.4 ≤ K < 4.7**	1.12 (1, 1.25)	0.060	1.16 (1.02, 1.32)	0.022	0.97 (0.74, 1.27)	0.828
**4.7 ≤ K ≤ 5.5**	1 (ref)		1 (ref)		1 (ref)	
**rs5219**	0.99 (0.93, 1.06)	0.751	0.99 (0.92, 1.06)	0.715	1.02 (0.83, 1.26)	0.828
**Fully Adjusted, Model 3**						
**Serum K (mmol/L)**						
**2.5 ≤ K < 4.1**	1.21 (1.05, 1.4)	0.008	1.18 (0.99, 1.41)	0.062	1.2 (0.92, 1.58)	0.187
**4.1 ≤ K < 4.4**	1.3 (1.15, 1.47)	0.000	1.25 (1.08, 1.45)	0.002	1.29 (0.99, 1.68)	0.063
**4.4 ≤ K < 4.7**	1.14 (1.01, 1.3)	0.033	1.15 (1, 1.32)	0.046	1 (0.75, 1.34)	0.997
**4.7 ≤ K ≤ 5.5**	1 (ref)		1 (ref)		1 (ref)	
**rs5215**	1.01 (0.94, 1.08)	0.843	1.02 (0.94, 1.1)	0.663	0.93 (0.76, 1.15)	0.511
**rs5219**	1.02 (0.95, 1.1)	0.590	1.01 (0.94, 1.09)	0.741	1.08 (0.86, 1.36)	0.520
**Fully Adjusted, Model 4 with both K and SNP**						
**Serum K (mmol/L)**						
**2.5 ≤ K < 4.1**	1.21 (1.05, 1.4)	0.008	1.18 (0.99, 1.41)	0.062	1.2 (0.92, 1.57)	0.187
**4.1 ≤ K < 4.4**	1.3 (1.15, 1.47)	0.000	1.25 (1.09, 1.45)	0.002	1.29 (0.99, 1.68)	0.063
**4.4 ≤ K < 4.7**	1.14 (1.01, 1.3)	0.033	1.15 (1, 1.32)	0.045	1 (0.75, 1.34)	1.000
**4.7 ≤ K ≤ 5.5**	1 (ref)		1 (ref)		1 (ref)	
**rs5215**	1.01 (0.94, 1.08)	0.819	1.02 (0.94, 1.1)	0.638	0.93 (0.76, 1.14)	0.505
**2.5 ≤ K < 4.1**	1.21 (1.05, 1.4)	0.008	1.18 (0.99, 1.41)	0.062	1.2 (0.92, 1.58)	0.183
**4.1 ≤ K < 4.4**	1.3 (1.15, 1.47)	0.000	1.25 (1.09, 1.45)	0.002	1.29 (0.99, 1.68)	0.063
**4.4 ≤ K < 4.7**	1.14 (1.01, 1.3)	0.032	1.15 (1, 1.32)	0.045	1 (0.75, 1.34)	0.998
**4.7 ≤ K ≤ 5.5**	1 (ref)		1 (ref)		1 (ref)	
**rs5219**	1.02 (0.95, 1.1)	0.571	1.01 (0.94, 1.1)	0.717	1.08 (0.86, 1.36)	0.509

**Model 1-** adjusted for age, sex, race (overall only), SNPs and serum K analyzed in separate models

**Model 2-** adjusted for age, sex, race (overall only), SNP (rs5215 or rs5219), K

**Model 3-** adjusted for age, sex, race (overall only), BMI, systolic blood pressure, presence of hypertension, use of ABD medications, serum creatinine, serum sodium, fasting glucose, fasting insulin, parental history of diabetes mellitus, physical activity, income, SNPs and serum K analyzed in separate models

**Model 4-** adjusted for age, sex, race (overall only), BMI, systolic blood pressure, presence of hypertension, use of ABD medications, serum creatinine, serum sodium, fasting glucose, fasting insulin, parental history of diabetes mellitus, physical activity, income, SNP (rs5215 or rs5219), K

In the entire cohort, after adjusting for age, sex, and race, rs5215 was not significantly associated with odds of diabetes, with an OR (95% CI) of 0.98 (0.92, 1.05). In the minimally-adjusted model, stratified by race, similar results were obtained for whites and blacks. In the entire cohort, adjusting for age, sex, and race, rs5219 was also not associated with odds of diabetes, with an OR (95% CI) of 0.99 (0.92, 1.06); with no significant association for whites or blacks in stratified models (Model 1, Table 3). Results were similar for both serum K and each *KCNJ11* variant when both serum K and each variant were included in minimally-adjusted models (Model 2, Table 3).

### Models 3 and 4, fully-adjusted multivariable models

In multivariable models, serum K continued to be a significant predictor of incident diabetes both without adjustment for the *KCNJ11* variants of interest and with adjustment for the *KCNJ11* variants of interest (Models 3 and 4, Table 2). In the model adjusted for rs5215, those in the lowest quartile of serum K had higher odds of incident diabetes compared to those in the highest quartile of serum K, with an OR (95% CI) of 1.21 (1.05, 1.4). In the model adjusted for rs5219, compared to those in the highest quartile of serum K, those in the lowest quartile of serum K had an OR (95% CI) of incident diabetes of 1.21 (1.05, 1.4). In models stratified by race, this association between serum K and odds of incident diabetes persisted in whites but was no longer significant in blacks (Models 3, 4, Table 3).

In the multivariable models both with and without adjustment for serum K, neither rs5215 nor rs5219 was a significant predictor of incident diabetes (Model 3, 4, Table 3).

### Sensitivity analyses

In multivariable models, with serum K as a continuous value scaled by its standard deviation, serum K continued to have an inverse association with diabetes risk. In model 2 adjusted for rs5215 and the interaction term serum K X SNP, serum K had an OR (95% CI) of incident diabetes of 0.88 (0.83, 0.94); similarly, in model 2 adjusted for rs5219 and the interaction term serum K X SNP, the OR (95% CI) for serum K was 0.89 (0.84, 0.95) (**Table 4**).

**Table 4:** Associations of serum K (scaled/continuous), rs5215, and rs5219 with incident diabetes in 11,812 ARIC-JHS participants, overall and by race.

Minimally Adjusted, Model 1	Overall (n = 11,812)	P	Caucasians (n = 8653)	P	African Americans (n = 3159)	P
	OR (95% CI)		OR (95% CI)		OR (95% CI)	
**Serum K (mmol/L) (scaled)**	**0.86 (0.82, 0.89)**	**0.00**	**0.88 (0.83, 0.92)**	**0.00**	**0.82 (0.76, 0.88)**	**0.00**
**Fully Adjusted,** **Model 2 rs5215**						
**Serum K (mmol/L) (scaled)**	**0.88 (0.83, 0.94)**	**0.00**	**0.87 (0.8, 0.95)**	**0.00**	**0.9 (0.82, 0.99)**	**0.03**
**rs5215**	0.67 (0.35, 1.3)	0.24	0.55 (0.25, 1.22)	0.14	0.49 (0.07, 3.2)	0.45
**Interaction** **(serum K X rs5215)**	1.04 (0.97, 1.11)	0.23	1.06 (0.98, 1.15)	0.13	1.07 (0.88, 1.31	0.50
**Fully-adjusted,** **Model 2 rs5219**						
**Serum K (mmol/L)** **(scaled)**	**0.89 (0.84, 0.95)**	**0.00**	**0.88 (0.81, 0.95)**	**0.00**	**0.9 (0.82, 0.99)**	**0.02**
**rs5219**	0.79 (0.4, 1.54)	0.49	0.6 (0.27, 1.32)	0.20	0.38 (0.04, 3.54)	0.40
**Interaction** **(serum K X rs5219)**	1.03 (0.96, 1.1)	0.45	1.05 (0.97, 1.14)	0.19	1.12 (0.88, 1.41)	0.35

**Model 1-** adjusted for age, sex, race (Overall only)

**Model 2-** adjusted for age, sex, race (Overall only), BMI, systolic blood pressure, presence of hypertension, use of ABD medications, serum creatinine, serum sodium, fasting glucose, fasting insulin, parental history of diabetes mellitus, physical activity, income, serum K (quartiles), SNP (rs5215 or rs5219), and interaction terms (SNP X K)

In our combined cohort, when modeling rs5215 and rs5219 as dichotomized variables (carriers or non-carriers), neither variant was a significant predictor of incident diabetes. In multivariable model 4, rs5215 and rs5219 had OR (95% CI) of incident diabetes of 0.62 (0.26, 1.50) and 0.76 (0.31, 1.84), respectively.

In sensitivity analyses, we also separated our combined cohort into ARIC and JHS sub-groups. In the JHS sub-group, we adjusted for relatedness using generalized estimating equations in SAS 9.3 (284 out of 862 JHS participants in our analysis related to another included participant through a known pedigree, maximum family size 13). We found that this analysis framework did not substantively change the association between serum K and diabetes risk; the SNP of interest and diabetes risk; or the significance of the interaction between serum K X SNP versus a simple logistic regression model. In each of the sub-groups, we additionally adjusted for ten ancestry principal component variables, and we found no significant change in the association between serum K and diabetes risk; the SNP of interest and diabetes risk; or the significance of the interaction between serum K X SNP.

We conducted a post-hoc power calculation to help determine what sample size would be needed to detect a significant association and interaction effects. We assumed a diabetes incidence of 0.27 with 3 controls to each case. We assumed allele frequencies from 0.15 to 0.60 to cover the allele frequencies in both Caucasians and African Americans. Additionally, we assumed a main effects odds ratio for each variant of 1.1, consistent with published data, and a main effects odds ratio of about 1.3 for a standard deviation of 0.5 (from Tables 1 and 2). With these assumptions and with our sample size, we would have had good power to detect a serum K X SNP interaction effect as low as 1.15 in the full data set. However, we likely did not have adequate power to detect the interaction within race strata, especially among African Americans.

## Discussion

In these analyses of pooled data from ARIC and JHS, we confirmed that serum K is a predictor of incident diabetes, independent of traditional diabetes risk factors and independent of genetic variants of the *KCNJ11* gene. We found similar associations among Caucasians and African Americans, although, in stratified models, results were statistically significant only for whites. Additionally, in this combined cohort, we found that genetic variants of the *KCNJ11* gene are not significant predictors of diabetes risk for either race; and, these genetic variants are not effect modifiers of the association between serum K and incident diabetes for either race.

Serum K is an independent predictor of diabetes in ARIC, JHS, as well as in other cohorts, but not in all cohorts that have studied this association [1, 2, 19–21]. The potential biological mechanism explaining this association has not been definitively determined on a population level. However, the biological mechanism behind such an association can be hypothesized based on small clinical trials which have revealed that experimentally-induced hypokalemia (low serum K state) causes impairments in insulin secretion by pancreatic β-cells [5–7]. Similarly, the *KCNJ11* gene codes for ATP-sensitive K channels, which are instrumental in insulin secretion [8, 9]. These channels rely on a gradient of potassium across cellular membranes to allow entry of extracellular/serum K into cells, a high-K environment, which leads to channel closure, subsequent depolarization of the cell membrane, and stimulation of insulin secretion [9]. A higher serum K level, would allow for easier entry of K into the cells via these channels. Lower serum K would be less favorable for K entry into a high intracellular K environment, and thus may prevent adequate insulin secretion. One *in vitro* study has demonstrated the impact of different levels of extracellular K levels (equivalent to serum K) on the function of similar voltage-gated K channels [22]. Genetic variants of the ATP-sensitive K channels could, in theory, prevent entry of K into cells and be more sensitive to decreased K gradients across the cellular membranes. However, the function of these ATP-sensitive K channels is complex and could be affected by other factors as well. Another *in vitro* study found that *KCNJ11* variants led to decreased binding of ATP which led to impaired channel closure, thereby impairing insulin secretion [23]. This type of functional change would likely not result in sensitivity of the channel to K gradients.

While the association between serum K and diabetes risk was not found to be modified by the presence of the *KCNJ11* variants, the risk of diabetes in certain sub-populations may be more influenced by serum K levels than others. Prior analyses of the ARIC cohort found that the association between serum K and diabetes risk was stronger among African Americans compared to Caucasians and that serum K was a significant mediator of the association between race and diabetes risk [3, 4].

However, in this analysis of a combined ARIC and JHS cohort, while we did find significant associations between serum K and diabetes risk among both Caucasians and African Americans, the associations in this current analysis were not stronger among African Americans. The cohort created for these analyses was different from the cohort created for prior analyses; participants were excluded for missing data of different covariates (particularly genetic data). Additionally the prior analyses of the ARIC cohort utilized Cox models, while these analyses use logistic regression, which can also account for some of the differences in effect sizes. Therefore, further study is needed to determine whether or not the influence of serum K on risk of diabetes is stronger in African Americans compared to whites.

Other cohorts have identified other sub-populations whose risk of diabetes may be more influenced by serum K than others. A retrospective cohort study of patients followed in an Israeli health care system found a significant inverse association between serum K and diabetes risk; but this study found that age modified the association between serum K and dysglycemia, with a significant inverse association only among patients ≤ 41 years old [21]. A cross-sectional study of a German cohort evaluated the association between serum K and new-onset diabetes as well as with prediabetes [24]. There was a significant inverse association between serum K and risk of new-onset diabetes; however, this inverse association was modified by the presence of hypertension and was significant only among those with hypertension. [24]. In this study, the multivariable models did include adjustment for use of blood pressure medications, including diuretics; however, there was no further detailed analysis of the influence of use of diuretics on this association of serum K and prediabetes among the participants with hypertension. Past studies have evaluated the association between antihypertensive medications and diabetes risk, and several studies, but not all, have found that use of thiazide diuretics is associated with increased glucose levels and diabetes risk [25–28]. Further study of clinical trials evaluating thiazide use has revealed that serum K, which decreases with use of thiazides, may mediate the association between thiazide use and diabetes risk [29, 30]. Based on the studies above, it is clear that further investigation is needed to determine the characteristics of people, whether demographic, anthropometric, clinical, or even genetic factors other than *KCNJ11* variants, whose diabetes risk could most be affected by low-normal serum K.

The finding that the presence of *KCNJ11* variants was not a significant predictor of diabetes risk in this cohort was not completely unexpected. The significance of *KCNJ11* variants on diabetes risk in other cohorts has been evaluated, but these cohorts have primarily been Caucasian or Asian. A meta-analysis of the effects of *KCNJ11* on diabetes risk, combing results from 48 studies of Caucasian, East Asians and Indians, found that the adjusted odds of incident diabetes OR (95% CI) per risk allele was 1.12 (1.09, 1.16) [31]. Similar results were observed in a meta-analysis of only Caucasian cohorts [32]. One meta-analysis assessed the significance of the presence of *KCNJ11* rs5215 among African Americans with similar results [adjusted OR (95% CI) of incident diabetes of 1.16 (1.09, 1.23)]. However, not all studies have found *KCNJ11* to be associated with increased diabetes risk. In the Diabetes Prevention Program (DPP), a cohort of US participants (n = 3,534) with impaired glucose tolerance at baseline and followed for incident diabetes, presence of *KCNJ11* variants was associated with decreased insulin secretion at baseline; but, over the initial 3 years of follow-up, the presence of these variants was not associated with an increased risk of diabetes [33]. Effect size heterogeneity or lack of power may contribute to null associations with incident diabetes risk, particularly in smaller cohorts.

In general, genetic studies have found that the presence of individual genetic risk variants may not confer a risk of diabetes that is significant, especially compared to clinical characteristics. However, several gene variants taken together may explain a higher proportion of diabetes risk; and in aggregate, may help identify patients that would benefit from a greater emphasis on prevention efforts.

While this study has several strengths, including a multi-ethnic population with data on multiple covariates, several limitations of these analyses should be mentioned, particularly with relation to the size of our combined cohort. Generalizability of this study may be limited by the following: a significant portion of the cohort (11%) did not have genetic data available; others were excluded due to missing information on variables; and these results are from two US community-based studies, with most of the African Americans primarily from one site, Mississippi, which is an area with characteristics and disease risks which are different than other areas in the US. Finally, we selected the ARIC and JHS cohorts for these analyses because of our past studies, which showed a significant association between serum K and diabetes risk. However, this sample size of this combined cohort is likely under-powered, particularly based on our post-hoc power calculation described in the results section, to detect significant associations between *KCNJ11* variants and diabetes risk and to detect potential interactions of serum K with the *KCNJ11* variants. Also, we did not adjust our analysis for ancestry principal components derived in a combined JHS and ARIC cohort, though sub-group analyses with adjustment for JHS and ARIC specific ancestry principal components showed no impact on the observed associations. In future studies, evaluating these associations in a larger cohort with standardized principal component variables and adjusting for racial admixture may help to make estimates of associations more reliable, particularly for associations by race.

While this study of the combined ARIC-JHS cohorts found that *KCNJ11* variants are not significant risk factors for diabetes, our findings confirm the previously noted association between serum K and diabetes risk, independent of the presence of the *KCNJ11* variants. This study also found that this association is not modified by the presence of either of the two *KCNJ11* variants analyzed. Further study is needed to determine the characteristics, both clinical and genetic, of patients that may be at highest risk of diabetes due to a low-normal serum K; and to determine if serum K might be a modifiable risk factor for those high-risk populations.
